# Psychometric Tests and Spatial Navigation: Data From the Baltimore Longitudinal Study of Aging

**DOI:** 10.3389/fneur.2020.00484

**Published:** 2020-06-11

**Authors:** Eric X. Wei, Eric R. Anson, Susan M. Resnick, Yuri Agrawal

**Affiliations:** ^1^Department of Otolaryngology-Head and Neck Surgery, Johns Hopkins University School of Medicine, Baltimore, MD, United States; ^2^Department of Otolaryngology, University of Rochester Medical Center School of Medicine and Dentistry, Rochester, NY, United States; ^3^Laboratory of Behavioral Neuroscience, National Institute on Aging, Baltimore, MD, United States

**Keywords:** spatial cognition, visuospatial ability, triangle completion task, spatial navigation, aging

## Abstract

Spatial cognition is the process by which individuals interact with their spatial environment. Spatial cognition encompasses the specific skills of spatial memory, spatial orientation, and spatial navigation. Prior studies have shown an association between psychometric tests of spatial ability and self-reported or virtual measures of spatial navigation. In this study, we examined whether psychometric spatial cognitive tests predict performance on a dynamic spatial navigation task that involves movement through an environment. We recruited 151 community-dwelling adult participants [mean (*SD*) age 69.7 (13.6), range 24.6–93.2] from the Baltimore Longitudinal Study of Aging (BLSA). Spatial navigation ability was assessed using the triangle completion task (TCT), and two quantities, the angle and distance of deviation, were computed. Visuospatial cognitive ability was assessed primarily using the Card Rotations Test. Additional tests of executive function, memory, and attention were also administered. In multiple linear regression analyses adjusting for age, sex, race, and education, cognitive tests of visuospatial ability, executive function, and perceptual motor speed and integration were significantly associated with spatial navigation, as determined by performance on the TCT. These findings suggest that dynamic spatial navigation ability is related to spatial memory, executive function, and motor processing speed.

## Introduction

Spatial cognition is the domain of cognitive function that relates to the processing of information about one's spatial environment. Spatial cognition encompasses the specific skills of spatial orientation (including mental rotation), spatial memory, and spatial navigation. Numerous studies have documented declines in spatial cognition associated with age (1–3), and studies suggest that spatial cognition is among the earliest domains of cognitive function to show impairment during the transition from normal cognitive aging to Alzheimer's disease (4). Impaired spatial cognition has been linked to functional limitations and adverse outcomes in older adults, including driving difficulty, losing, or misplacing objects, difficulty navigating new environments, and falls (5–11).

Static psychometric measures of spatial orientation (and mental rotation), such as the Card Rotations Test, are sometimes used as a proxy in clinical and research settings for assessing spatial cognitive abilities (12, 13). However, it is unclear how these stationary tests of spatial memory and orientation relate to dynamic spatial cognitive abilities and moreover, whether or not these tests can predict spatial navigation abilities at the scale of environments in which humans interact and move through. In particular, path integration is a critical spatial navigational strategy employed by humans whereby individuals use self-motion cues (e.g., vestibular sensory input and proprioceptive feedback) to maintain a sense of position and orientation. Although it has been shown that psychometric tests of spatial ability are predictive of performance on a virtual maze task (12), it is unclear whether these paper and pencil-based tests are able to predict performance on path integration tasks that require actual movement through space.

In this study, we used data from the Baltimore Longitudinal Study of Aging (BLSA) to assess the cross-sectional association between static psychometric tests that tap into spatial memory and orientation, and spatial navigation as determined by performance on the Triangle Completion Task (TCT). Individual differences in spatial navigational abilities, mediated by a combination of genetic, and environmental influences, are known to be particularly prominent at later stages of life (14), which supports the use of an aging cohort sensitive to variations in individual navigational abilities. The TCT is a dynamic test of spatial navigation that requires participants to create and retain a spatial map of their traveled path using self-motion cues, a process known as path integration (15–18). We hypothesized that visuospatial ability as measured by the Card Rotations Test would be associated with path integration as measured by performance on the TCT. We also examined the relationship between other domains of cognitive function, specifically verbal and non-verbal memory, executive function, language fluency, attention, and visuo-motor scanning, perceptual motor speed and integration, and general mental status, and performance on the TCT. These analyses provide insight into spatial memory, orientation and navigation as closely related vs. discrete abilities, and whether standard psychometric tests reflect path integration ability.

## Materials and Methods

### Study Population

The BLSA is a prospective cohort study followed by the National Institute on Aging (NIA) Intramural Research Program in Baltimore, Maryland. The data are available to the public via the BLSA Investigator's portal. Analysis code will be shared by the corresponding author to other researchers upon request. In this study, a cross-sectional sample of BLSA participants were evaluated who underwent TCT testing and cognitive function testing from January 2016 to March 2017. Participants with cognitive impairment, as determined by Mini-Mental Status Examination (MMSE) score ≤ 24, were excluded. Participants were also asked if they were ever told by a doctor that they had osteoporosis, osteoarthritis, and/or spinal stenosis. All participants provided written informed consent, and the study protocol was approved by the institutional review board of the National Institute of Environmental Health Science, National Institutes of Health.

### Triangle Completion Task (TCT)

The TCT was developed as a test of egocentric navigation. Test procedures have been described previously in detail (18), and are briefly described here. In brief, participants were asked to walk four triangular paths (twice clockwise, twice counterclockwise) of a 30°-60°-90° configuration with dimensions of 92.5 × 185.5 × 212 cm. Visual and auditory inputs were minimized or attenuated using a blindfold and noise-reducing headphones, respectively. Participants had to rely solely on vestibular, proprioceptive, and motor efference cues to complete the task. The examiner guided the participant through the first two segments including the 90-degree turn of the triangle and were asked to return back to the origin at the end of the second segment. The endpoint for each trial was determined as the midpoint between the anterior tips of the feet. The distance (in cm) between the endpoint and the starting point was averaged over the four trials and termed “distance of deviation.” Additionally, the difference between the absolute value of the angle the participant made at the end of the second limb of the triangle and the correct value of the corresponding angle was averaged over the four trials and termed “angle of deviation.”

### Cognitive Function Tests

Trained, certified examiners performed psychometric testing in the BLSA. The test battery assesses a number of cognitive domains, including verbal memory (California Verbal Learning Test), non-verbal memory (Benton Visual Retention Test), visuospatial ability (Card Rotations Test), executive function (TMT-B, Backward Digit Span, Digit Symbol Substitution Test), language fluency, and executive function (letter and category fluency), attention and visuo-motor scanning (TMT-A, TMT-B, Forward Digit Span), perceptual motor speed and integration (Purdue Pegboard), psychomotor speed (Digit Symbol Substitution Test), and mental status (Mini-Mental State Examination). The procedures for the battery of tests have been published in detail (19), and are briefly described here.

#### California Verbal Learning Test (CVLT)

The CVLT evaluates verbal learning and memory (20). In this test, a list of 16 shopping list items are read five times, with participants asked to recall the items after each repetition (immediate recall) and then again after 20 min (delayed recall).

#### Benton Visual Retention Test (BVRT)

The BVRT evaluates non-verbal memory and visuoconstructional skill. In this test, a card with an image of a geometric shape is shown for 10 s. When the card is removed, the participant is asked to draw the shape on a blank piece of paper. The number of errors was measured over 10 trials with 10 different cards (21).

#### Card Rotations Test

The card rotations test evaluates primarily visuospatial ability. In this test, a reference 2-dimensional geometric shape is shown to the participants. Following, a set of similar 2-dimensional objects are shown, with participants being asked to mentally rotate each object and determine whether it is identical or a mirror image of the reference object. The outcome of interest was the number of correctly classified objects minus the number of incorrectly classified objects (22).

#### Trail Making Test Parts A and B (TMT-A and TMT-B)

The TMT-A and TMT-B evaluate attention, processing speed, and visual scanning ability, while the TMT-B also evaluates executive function. In the TMT-A, 25 circles numbered 1–25 are distributed in a random order over a sheet, with participants being asked to draw a trail to connect the numbers in consecutive order (1, 2, 3, etc.) as quickly as possible while maintaining accuracy. In the TMT-B, similarly there are 25 circles with some containing numbers (1–13) and some containing letters (A-L). Participants are asked to draw a trial to connect the numbers and letters in alternating consecutive order (1, A, 2, B, etc.). The outcome of interest was the time to complete each task (23).

#### Digit Symbol Substitution Test (DSST)

The DSST evaluates executive function, visuospatial ability, and processing speed. Participants are shown nine pairs of digits and symbols, followed by a series of digits, in which participants are asked to draw the correct symbol that matches to each digit in the series. The outcome of interest is the number of correct digit-symbol matches made in 90 s (24).

#### Category and Letter Fluency Tests

The Category and Letter Fluency Tests evaluate language fluency and executive function. In the Category Fluency test, participants are given a category (animals, fruits, and vegetables) and asked to recite as many words as possible in 1 min that belong in that category. Similarly, in the Letter Fluency test, participants are given a letter (F, A, and S) and asked to recite as many words as possible that begin with that letter. The outcome of interest was the mean number of words recited across the three trials for each test (25).

#### Forward and Backward Digit Span Test

The digit span test from the Wechsler Adult Intelligence Scale–Revised contains both a forward recall component and a backward recall component. Both components evaluate attention and short-term memory while backward recall also evaluates mental manipulation and executive function. In this test, an increasingly longer lists of digits are recited to the participant who must recall each list immediately afterwards (forwards or backwards), until the participant is no longer able to accurately recall the digits. The outcome of interest is the maximum number of digits recalled correctly (26).

#### Purdue Pegboard Test

The Purdue Pegboard Test evaluates visuomotor integration as well as manual dexterity of both the dominant and non-dominant hand. Participants are shown a board with small holes and a cup filled with pegs. Participants are asked to insert as many pegs into the board as possible over 30 s using one hand. The outcome of measure is the mean number of pegs placed into the board over two trials with each hand (dominant and non-dominant hands). The mean between the two hands is also calculated (27).

#### Mini–Mental State Examination

The MMSE evaluates global mental status and is utilized in clinical settings to screen for cognitive impairment and dementia (28). It has a maximum score of 30 points. Participants with MMSE scores of 24 or lower were excluded from the analyses.

### Proprioception Testing

Ankle proprioception threshold testing has been previously validated and described in detail (29). In brief, participants, while in a seated position, placed their right foot on a motorized pedal. Participants indicated perception of ankle motion by pressing a button while blindfolded. The test examined the minimal angular displacement (i.e., threshold) required for the participant to recognize passive movement of the ankle joint at an angular speed of 0.3°/s. Testing followed a pre-set sequence of ankle plantar flexion, dorsiflexion, dorsiflexion, and plantar flexion. The proprioception threshold was determined to be the average angular displacement between the last two trials.

### Vestibular Evoked Myogenic Potential (VEMP) Testing

Air-conduction evoked cervical VEMP (cVEMP) was performed to assess saccular function. The procedure has previously been described in detail (30). In brief, participants were reclined at 30° from horizontal. Tonic background sternocleidomastoid (SCM) activity was elicited by having participants turn their heads to the right and left. Air-conducted positive polarity tone bursts war delivered monaurally at 500 Hz and 125 dB SPL through a noise-excluding headset. Electromyographic (EMG) signals were recorded with Ag/AgCl electrodes from GN Otometrics (Schaumburg, IL, USA) using a commercial EMG system (Carefusion Synergy, version 14.1, Dublin, OH, USA). An absent cVEMP response was defined as EMG recordings lacking definable p13 waves. Normal saccular function was defined as the presence of a vestibular evoked myogenic potential bilaterally. Abnormal saccular function was defined as either unilaterally or bilaterally absent function.

### Statistical Analysis

The main outcome of interest in this study was performance on the TCT. The main predictor variables of interest were performance on cognitive function tests. Descriptive analyses were conducted to examine participant demographics and performance on cognitive function tests. Multiple linear regression models adjusting for demographic characteristics (age, sex, race, and education) were developed to explore the association between performance on each cognitive function test and distance and angle of deviation on the TCT. Postregression estimates and plots were carried out to check that the requirements of multiple linear regression analyses were met. Augmented component-plus-residual plots and Kernel density plots were evaluated to ensure that there was a roughly linear relationship between cognitive variables and TCT performance and an approximately normal distribution of residuals, respectively. Variance inflation factors were calculated to rule out multicollinearity. Cook's distance was used to detect highly influential data points for each regression model, allowing for the identification of a single observation that was considered a highly influential data point in a majority of regression models involving both angle of deviation and distance of deviation on the TCT. Sensitivity analyses conducted to evaluate the impact of excluding this outlier showed a marginal difference in multiple regression analyses, so the participant was not excluded from the final analyses.

Exploratory factor analysis was performed to identify latent cognitive abilities underlying the battery of cognitive tests. Eigenvalues > 0.8 was used as a cutoff to determine the factors to retain. Multiple linear regression analyses were conducted to assess the association between factors identified and distance and angle of deviation on the TCT. A *p* < 0.05 was considered statistically significant. All analyses were performed using STATA version 14 (College Station, TX, USA).

## Results

The study population consisted of 151 participants with a mean age of 69.7 +/– 13.6 years (Table 1); 44.4% were female and 65.6% were white. Most participants (83.4%) had a college education or greater. A limited number of participants had missing responses on one or more cognitive function tests; the mean performance of the study sample on each of the cognitive function tests are presented in Table 1.

**Table 1:** Demographic characteristics and results of cognitive testing in the Baltimore Longitudinal Study of aging (*N* = 151).

	***N***	**Mean (*SD*)**	***N* (%)**
**Sex**	151		
Male			84 (55.6%)
Female			67 (44.4%)
**Mean age (*****SD*****)**	151	69.7 (13.6)	
**Race**	151		
White			99 (65.6%)
Non-white			52 (34.4%)
**Education**	151		
Less than college			25 (16.6%)
College			40 (26.5%)
Greater than college			86 (57.0%)
**Learning and memory:**			
Verbal: California verbal learning test			
Immediate recall	149	51.8 (11.1)	
Delayed recall	149	10.8 (3.3)	
Figural/non-verbal			
BVRT, errors	151	10.0 (5.8)	
**Visuospatial ability**			
Card rotations test^a^	145	88.7 (42.4)	
**Executive function**			
TMT-B, seconds^a^^,^ ^b^	148	74.1 (40.1)	
Backward digit span	151	7.0 (2.2)	
Digit symbol substitution test^a^^,^ ^c^	144	44.6 (12.6)	
**Language fluency and executive function**			
Letter fluency, mean	150	15.1 (4.0)	
Category fluency, mean	150	15.9 (3.5)	
**Attention and visuo-motor scanning**			
TMT-A, seconds^a^	151	30.5 (11.6)	
Forward digit span	151	8.2 (2.5)	
**Perceptual motor speed and integration**			
Purdue pegboard^a^	
Dominant	148	12.8 (2.2)	
Non-dominant	148	12.4 (2.1)	
Mean	147	12.6 (2.0)	
**Mental status**			
MMSE	150	28.7 (1.3)	

a *Tests which have a perceptual motor speed component*.

b *TMT-B also has an attention and visuo-motor scanning component*.

c *Digit symbol substitution test also has a psychomotor speed component*.

The association between cognitive function testing and deviation on the TCT was evaluated using simple linear regression models (Table 2) and multiple linear regression models controlling for age, sex, race, and education (Table 3). To facilitate interpretation, cognitive test scores were converted into standardized variables (i.e., z-scores) and the model coefficients were transformed such that a negative coefficient always indicated that better cognitive function was associated with decreased distance or angle of deviation on the TCT. In multiple linear regression analyses, distance of deviation on the TCT was significantly associated with visuospatial ability as determined by the Card Rotations test (β −5.9, *p* = 0.03). Additionally, distance of deviation was significantly associated with tests of executive function, namely the TMT-B (β −5.9, *p* = 0.04) and the DSST (β −10.9, *p* = 0.002), as well as tests of perceptual motor speed and integration, namely the Purdue Pegboard-dominant hand (β −9.2, *p* = 0.01) and the Purdue Pegboard-mean (β −8.3, *p* = 0.02). Notably, the psychometric test demonstrating the strongest relationship with both angular and distance deviation on the TCT was the DSST. Additionally, significant associations were found between angle of deviation on the TCT and performance on the DSST (β −3.4, *p* = 0.01). Performance on the TCT (either distance or angle of deviation) was not significantly associated with verbal or non-verbal memory tests (CVLT and BVRT), language fluency (letter and category fluency), attention and visuo-motor scanning (TMT-A, forward digit span), or global mental status (MMSE) in multiple linear regression models. See Supplementary Tables 1–15 for coefficients and p-values of other covariates in the multiple regression models. After adjusting for presence of orthopedic issues (osteoarthritis, osteoporosis, and spinal stenosis) and ankle proprioception thresholds, there were no substantial changes in these findings (results not shown). Additionally, to evaluate whether vestibular function might be a confounder in the relationship between performance on psychometric tests and the TCT, we added the cervical vestibular evoked myogenic potential (cVEMP) into the six regression models that demonstrated a significant relationship between performances on psychometric tests and the TCT. In previously published work examining the relationship between vestibular function and the TCT, we found that saccular function, as measured by the cVEMP was significantly associated with both angular and distance errors on the TCT in healthy older adults (30). Here, we found that after adjusting for vestibular function, the relationship between psychometric measures and the TCT was not substantially attenuated in five out of six of the regression models; only the relationship between the Card Rotations test and distance of deviation on the TCT was mildly attenuated and no longer significant after adding cVEMP to the regression model (from β −5.9, *p* = 0.03 to β −5.1, *p* = 0.08).

**Table 2:** Simple linear regression of deviation on the triangle completion task and cognitive function tests in the Baltimore Longitudinal Study on aging.

**Cognitive test**	**Distance of deviation (cm)**			**Angle of deviation**		
	**β (95% CI)^a^^,^^b^**	***p*-value**	***R*^**2**^**	**β (95% CI) ^a^^,^^b^**	***p*-value**	***R*^**2**^**
**Learning and memory:**						
Verbal: California verbal learning test						
Immediate recall	−2.6 (−7.9, 2.6)	0.32	0.01	−1.4 (−3.2, 0.5)	0.16	0.01
Delayed recall	−1.4 (−6.6, 3.9)	0.6	0.002	−0.9 (−2.8, 1.0)	0.34	0.01
Figural/non-verbal						
BVRT, errors	**−5.7 (−10.9**, **−0.4)**	**0.03**	**0.03**	**−1.9 (−3.7**, **−0.01)**	**0.05**	**0.03**
**Visuospatial ability**						
Card rotations test	**−8.3 (−13.5**, **−3.2)**	**0.002**	**0.07**	**−2.4 (−4.3**, **−0.5)**	**0.01**	**0.04**
**Executive function**						
TMT-B, seconds	**−7.2 (−12.5**, **−2.0)**	**0.007**	**0.05**	**−2.3 (−4.2**, **−0.5)**	**0.01**	**0.04**
Backward digit span	−2.5 (−7.8, 2.8)	0.36	0.01	−0.8 (−2.7, 1.1)	0.39	0.005
Digit symbol substitution test	**−12.5 (−17.4**, **−7.5)**	**<0.001**	**0.15**	**−4.0 (−5.9**, **−2.2)**	**<0.001**	**0.12**
**Language fluency and executive function**						
Letter fluency, mean	**−5.2 (−10.4**, **−0.02)**	**0.05**	**0.03**	**−2.0 (−3.9**, **−0.2)**	**0.03**	**0.03**
Category fluency, mean	**−8.3 (−13.4**, **−3.3)**	**0.001**	**0.07**	**−3.1 (−5.0**, **−1.3)**	**0.001**	**0.07**
**Attention and visuo-motor scanning**						
TMT-A, seconds	**−5.7 (−10.9**, **−0.4)**	**0.03**	**0.03**	−1.8 (−3.6, 0.1)	0.06	0.02
Forward digit span	−3.6 (−8.9, 1.7)	0.18	0.01	01.1 (−3.0, 0.7)	0.23	0.01
**Perceptual motor speed and integration**						
Purdue pegboard						
Dominant	**−11.4 (−16.4**, **−6.3)**	**<0.001**	**0.12**	**−3.2 (−5.0**, **−1.4)**	**0.001**	**0.08**
Non-dominant	**−9.0 (−14.2**, **−3.8)**	**0.001**	**0.07**	**−2.8 (−4.6**, **−0.9)**	**0.003**	**0.06**
Mean	**−10.8 (−15.9**, **−5.7)**	**<0.001**	**0.11**	**−3.2 (−5.0**, **−1.3)**	**0.001**	**0.07**
**Mental status**						
MMSE	−3.8 (−9.1, 1.5)	0.16	0.01	−1.2 (−3.0, 0.7)	0.23	0.01

a *Unadjusted model*.

b *Standardized Regression Coefficients were used. Negative β coefficients indicate decreased distance of deviation with greater cognitive function*.

*The bolded values are significant (as determined by p-value < 0.05)*.

**Table 3:** Multiple linear regression of deviation on the triangle completion task and cognitive function tests in the Baltimore Longitudinal Study on aging.

**Cognitive test**	**Distance of deviation (cm)**			**Angle of deviation**		
	**β (95% CI)^a^^,^^b^**	***p*-value**	***R*^**2**^**	**β (95% CI) ^a^^,^^b^**	***p*-value**	***R*^**2**^**
**Learning and memory:**						
***Verbal: California verbal learning test***						
Immediate recall	2.4 (−3.4, 8.1)	0.42	0.16	0.4 (−1.7, 2.5)	0.71	0.12
Delayed recall	2.4 (−3.1, 7.8)	0.39	0.16	0.5 (−1.5, 2.5)	0.61	0.12
***Figural/non-verbal***						
BVRT, errors	−3.0 (−8.8, 2.8)	0.31	0.15	−0.8 (−2.8, 1.3)	0.47	0.12
**Visuospatial ability**						
Card rotations test	**−5.8 (−11.4**, **−0.2)**	**0.03**	**0.18**	−1.6 (−3.7, 0.5)	0.12	0.13
**Executive function**						
TMT-B, seconds	**−5.9 (−11.3**, **−0.4)**	**0.04**	**0.18**	−1.6 (−3.5, 0.4)	0.12	0.14
Backward digit span	−2.0 (−7.3, 3.3)	0.46	0.14	−0.6 (−2.5, 1.3)	0.53	0.12
Digit symbol substitution test	**−10.9 (−17.7**, **−4.0)**	**0.002**	**0.21**	**−3.4 (−5.9**, **−0.8)**	**0.01**	**0.15**
**Language fluency and executive function**						
Letter fluency, mean	−4.8 (−10.0, 0.4)	0.07	0.17	−1.7 (−3.6, 0.2)	0.08	0.13
Category fluency, mean	−4.1 (−10.5, 2.3)	0.21	0.16	−1.7 (−4.1, 0.6)	0.15	0.13
**Attention and visuo-motor scanning**						
TMT-A, seconds	−2.5 (−7.9, 3.0)	0.37	0.15	−0.6 (−2.5, 1.4)	0.57	0.12
Forward digit span	−2.6 (−7.9, 2.7)	0.34	0.15	−1.0 (−2.9, 0.9)	0.32	0.12
**Perceptual motor speed and integration**						
Purdue pegboard						
Dominant	**−9.2 (−16.1**, **−2.3)**	**0.01**	**0.18**	−1.6 (−4.1, 1.0)	0.22	0.12
Non-dominant	−5.1 (−12.0, 1.8)	0.14	0.16	−1.1 (−3.6, 1.4)	0.39	0.12
Mean	**−8.3 (−15.5**, **−1.1)**	**0.02**	**0.17**	−1.5 (−4.2, 1.1)	0.25	0.12
**Mental status**						
MMSE	−2.2 (−7.5, 3.2)	0.42	0.15	−0.3 (−2.2, 1.6)	0.76	0.12

a *Model adjusted for age, sex, race, and education*.

b *Standardized Regression Coefficients were used. Negative β coefficients indicate decreased distance of deviation with greater cognitive function*.

*The bolded values are significant (as determined by p-value < 0.05)*.

Exploratory factor analysis was performed to identify latent cognitive abilities underlying the battery of cognitive tests. One hundred and thirty-four (88.7%) out of 151 participants had complete data on all cognitive tests and contributed to the factor analysis. Factor analysis yielded three factors, which based on the loading structure of the cognitive outcomes, were defined as visuospatial ability, verbal memory, and working memory and attention. The loading structure of the cognitive outcomes on these three factors was similar to the loading structure reported in a previous study that used the same battery of cognitive tests (19). Rotated factor loadings are shown in Supplementary Table 16. The visuospatial ability factor and working memory and attention factor were significantly positively correlated (*r* = 0.88, *p* < 0.001). Meanwhile, the verbal memory factor was not significantly correlated with either the visuospatial factor (*r* = 0.02, *p* = 0.79) or the working memory and attention factor (*r* = −0.04, *p* = 0.61). We conducted multivariate analyses controlling for demographic factors to examine the relationship between each of the three factors individually and performance on the TCT. The visuospatial factor was significantly associated with distance of deviation (β = −1.30, *p* < 0.001) and angle of deviation (β = −0.40, *p* = 0.002) on the TCT. Similarly, the working memory and attention factor was also significantly associated with distance of deviation (β = −0.85, *p* = 0.003) and angle of deviation (β = −0.28, *p* = 0.010) on the TCT. The verbal memory factor was not significantly associated with either distance of deviation or angle of deviation on the TCT. When all three factors were included in a single model along with demographic factors, the visuospatial factor was significantly related to distance of deviation (β = −1.86, *p* = 0.014) and nearly significantly related to angle of deviation (β = −0.50, *p* = 0.085) on the TCT. Notably, neither the working memory and attention factor or the verbal memory factor were significantly related to performance on the TCT in this model.

## Discussion

In this study of healthy adults, we observed that cognitive tests of visuospatial ability, as well as executive function and perceptual motor speed and integration were significantly associated with path integration, as determined by performance on the TCT. Spatial navigation is the fundamental process by which humans and other organisms estimate their position in space and track their movement through their environment. Navigation strategies include allocentric navigation, i.e., orientation and movement are calculated relative to visual landmarks and external cues, and egocentric navigation, i.e., individuals use self-motion cues provided by the visual system, vestibular system, and proprioception to track their movement (a process known as path integration) (31–33). Prior studies including from the BLSA have shown a decline in allocentric navigation in a virtual environment with age (34), and have also shown a link between allocentric spatial navigation ability in a virtual environment and measures of mental rotation and verbal and visual memory (35). It has also been demonstrated that path integration ability is also reduced in older relative to younger adults (18), and aging may specifically impair the ability to switch from an egocentric to an allocentric navigational strategy (36). In this study we provide evidence that psychometric measures of visuospatial ability as well as executive function and perceptual motor speed and integration are related to path integration skills.

The appropriateness of using psychometric tests of visuospatial ability as a predictor of spatial navigational abilities has been debated. Whereas, studies of spatial cognition in animals have typically employed maze-learning tasks such as the Morris water navigation task which require actual movement through space, studies of spatial cognition in humans have traditionally employed paper-and-pencil tests of visuospatial ability (12). Indeed, it has been argued that subjective self-reported sense of direction may be a better predictor of spatial navigational abilities than performance on psychometric tests of visuospatial ability, as these tests fail to provide or require self-motion cues, a necessary feature of path integration (13). However, the current findings are more consistent with and build on previous studies that support an association between psychometric tests of visuospatial ability and spatial navigation. One study found highly significant correlations between scores on psychometric tests of spatial ability and performance on a virtual maze test, which simulates visual sensory input and transformations, but not vestibular or proprioceptive information (12). Our findings extend these previous observations by demonstrating that the Card Rotations Test, a psychometric test of visuospatial ability is associated with performance on a blinded path integration task in which vestibular and proprioceptive sensory inputs were available. Furthermore, in factor analyses, the visuospatial factor was most strongly associated with TCT performance. Notably, despite observing a highly significant relationship between path integration and visuospatial ability as well as other cognitive domains, we note that the *R*^2^ values are low in both simple and multiple regression analyses, consistent with our understanding that path integration is a complex navigational strategy dependent on multiple factors. As such, psychometric tests alone may not be sufficient to predict path integration.

In addition to supporting the association between psychometric tests of visuospatial ability and path integration, our study also demonstrates that path integration is associated with other psychometric measures, notably executive function, and perceptual motor speed and integration in multiple regression analyses. These findings build on an emerging body of evidence that have implicated the role of executive function and other cognitive domains in navigational ability. One study that used a modified TCT in which visual and proprioceptive inputs were removed via wheelchair during the first two legs of the TCT, found that older adults performed worse than younger adults, with 65% of the age-related variance in performance accounted for by performance on the Digit Symbol Substitution test, one of the same tests we used to evaluate executive function in this study (37). Another study in young adults found that performance on a virtual maze test was significantly associated with executive function including measures of inhibitory control and set switching (38). This aforementioned study also found that performance on the Category Fluency test, a measure that taps into language fluency and executive function, was significantly associated with navigational ability, although they did not adjust for important demographic covariates. Similarly, a study using a virtual-reality-based wayfinding task found that measures of executive function specifically working memory and inductive reasoning were significant predictors of performance on the task (39). The underlying mechanism driving the association between executive function and path integration may be related to the utilization of two different path integration strategies: configural vs. continuous. The configural strategy, which requires formation of a working memory representation of the traversed path, has been shown to be more accurate while the continuous strategy, which does not require forming a representation of the path traversed, may permit shorter response times (40). Taken in this context, it is possible that individuals with decreased executive function may be more likely to adopt a continuous navigational strategy over a configural one. Overall our findings build on these studies by demonstrating that associations between multiple cognitive domains and performance on a path integration task are present in a larger study sample and in multiple regression analyses.

Moreover, we found that visuospatial ability and perceptual motor speed were significantly associated with distance of deviation but not angle of deviation on the TCT in multiple regression analyses, while executive function was associated with both distance and angle of deviation. The angular error is made immediately upon ending the guided portion of the TCT and may thus represent immediate path integration ability whereas the distance error occurs at the end of the final segment of the triangle and may pose a greater demand for cognitive resources, specifically encoding spatial information into long-term memory. One study examining the relationship between age and performance on the TCT found that only distance of deviation was reliably correlated with age (37), suggesting that distance of deviation on the TCT may be a more sensitive marker of difficulty with path integration. Our findings thus support that mental rotation, may provide contributions to performance on at least one delayed measure of path integration in a large study sample of healthy adults.

We note several limitations of this study. Although we adjusted for important potential confounders of the association between psychometric tests and the TCT such as age, sex, race, and education level, there remains the possibility of confounding by unmeasured factors. Despite observing highly significant associations between cognitive variables and path integration, we note that the *R*^2^ values are low in multiple regression analyses. Future work is needed to evaluate the relative contributions of different cognitive domains, vestibular function, and proprioception on path integration. This population consisted of cognitively healthy adults and the results presented here may differ for individuals with cognitive impairment. Additionally, although angle and distance of deviation on the TCT were measured in this study, consistent with recent experiments using the TCT (18, 41), some studies have also measured error of the length of the hypotenuse (15, 37). An examination of the relationship of the hypotenuse measure on the TCT and cognitive function would be valuable in future studies.

In summary, we observed that performance in the cognitive domains of visuospatial ability, executive function, and perceptual motor speed and integration were significantly associated with spatial navigation ability, as determined by performance on the TCT. Despite the presence of associations, we observed that psychometric tests explained a relatively low level of the variation in performance on the TCT, suggesting that psychometric tests alone may not be sufficient to predict path integration. Future neuroimaging studies are needed to examine the neuroanatomic networks that may explain the link between psychometric measures of executive function and spatial memory and orientation and dynamic tests of spatial navigation.

## Data Availability Statement

Publicly available datasets were analyzed in this study. The data are available to the public via the Baltimore Longitudinal Study of Aging (BLSA) Investigator's portal.

## Ethics Statement

The studies involving human participants were reviewed and approved by National Institute of Environmental Health Science, National Institutes of Health. The patients/participants provided their written informed consent to participate in this study.

## Author Contributions

EW: substantial contribution to the conception and design of the work, analysis and interpretation of the data, drafting the manuscript and revising it critically for important intellectual content, final approval of the version to be published, and agreement to be accountable for the work. EA and YA: substantial contribution to the conception and design of the work, acquisition, analysis and interpretation of the data, revising the work critically for important intellectual content, final approval of the version to be published, and agreement to be accountable for the work. SR: substantial contribution to the conception and design of the work, analysis and interpretation of the data, revising the work critically for important intellectual content, final approval of the version to be published, and agreement to be accountable for the work.

## Conflict of Interest

The authors declare that the research was conducted in the absence of any commercial or financial relationships that could be construed as a potential conflict of interest.
