# Supplementary material for: Psychometric Tests and Spatial Navigation: Data From the Baltimore Longitudinal Study of Aging
Source: Front Neurol. 2020 Jun 11;11:484. doi: 10.3389/fneur.2020.00484 (PMC7300262; doi:10.3389/fneur.2020.00484)
Supplement: Supplementary file 1 [file Table_1.docx]

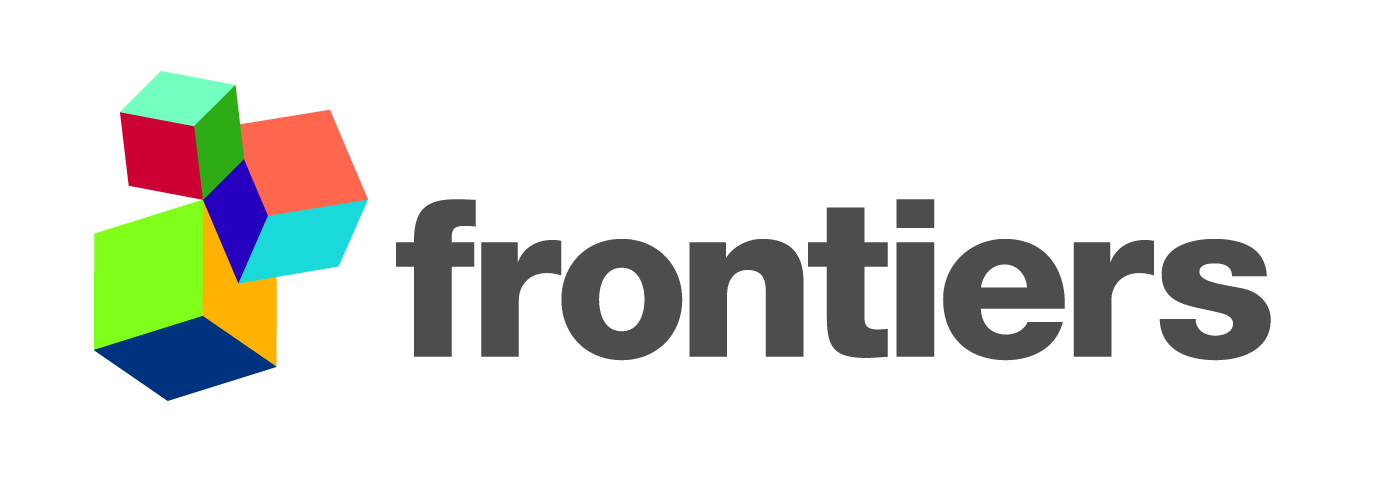


Supplementary Material

The role of psychometric tests in predicting spatial navigation ability: Data from the Baltimore Longitudinal Study of Aging

| **Supplementary Table 1: Multiple Linear Regression of Deviation on the Triangle Completion Task and California Verbal Learning Test (CVLT): Immediate Recall** | | | | |
| --- | --- | --- | --- | --- |
|  |  |  |  |  |
|  |  |  |  |  |
|  | **Distance of Deviation (cm)** | | **Angle of Deviation** | |
|  | **β (95% CI) ^a^** | **p-value** | **β (95% CI) ^a^** | **p-value** |
| CVLT: Immediate Recall | 2.4 (-3.4, 8.1) | 0.42 | 0.4 (-1.7, 2.5) | 0.71 |
| Age | **1.0 (0.5, 1.4)** | **<0.001** | **0.3 (0.1, 0.4)** | **<0.001** |
| Gender |  |  |  |  |
| Female | Reference |  | Reference |  |
| Male | -2.5 (-12.8, 7.8) | 0.63 | 0.9 (-2.9, 4.7) | 0.63 |
| Education |  |  |  |  |
| Less than college | Reference |  | Reference |  |
| College | 2.1 (-13.7, 17.8) | 0.80 | -1.9 (-7.7, 3.9) | 0.52 |
| Greater than college | 10.8 (-3.4, 24.9) | 0.13 | 1.7 (-3.5, 7.0) | 0.52 |
| Race |  |  |  |  |
| White | Reference |  | Reference |  |
| Non-white | 2.8 (-8.2, 13.7) | 0.62 | 0.9 (-3.1, 5.0) | 0.65 |

| **Supplementary Table 2: Multiple Linear Regression of Deviation on the Triangle Completion Task and California Verbal Learning Test (CVLT): Delayed Recall** | | | | |
| --- | --- | --- | --- | --- |
|  |  |  |  |  |
|  |  |  |  |  |
|  | **Distance of Deviation (cm)** | | **Angle of Deviation** | |
|  | **β (95% CI) ^a^** | **p-value** | **β (95% CI) ^a^** | **p-value** |
| CVLT: Delayed recall | 2.4 (-3.1, 7.8) | 0.39 | 0.5 (-1.5, 2.5) | 0.61 |
| Age | **0.9 (0.5, 1.3)** | **<0.001** | **0.3 (0.1, 0.4)** | **<0.001** |
| Gender |  |  |  |  |
| Female | Reference |  | Reference |  |
| Male | -2.6 (-12.8, 7.6) | 0.61 | 1.0 (-2.8, 4.7) | 0.62 |
| Education |  |  |  |  |
| Less than college | Reference |  | Reference |  |
| College | 1.6 (-14.3, 17.5) | 0.84 | -2.0 (-7.9, 3.8) | 0.49 |
| Greater than college | 10.7 (-3.5, 24.8) | 0.14 | 1.6 (-3.6, 6.9) | 0.54 |
| Race |  |  |  |  |
| White | Reference |  | Reference |  |
| Non-white | 2.2 (-8.5, 12.9) | 0.68 | 0.9 (-3.1, 4.8) | 0.66 |

| **Supplementary Table 3: Multiple Linear Regression of Deviation on the Triangle Completion Task and Benton Visual Retention Test (BVRT)** | | | | |
| --- | --- | --- | --- | --- |
|  |  |  |  |  |
|  |  |  |  |  |
|  | **Distance of Deviation (cm)** | | **Angle of Deviation** | |
|  | **β (95% CI) ^a^** | **p-value** | **β (95% CI) ^a^** | **p-value** |
| BVRT, errors | -3.0 (-8.8, 2.8) | 0.31 | -0.8 (-2.8, 1.3) | 0.47 |
| Age | **0.7 (0.3, 1.2)** | **0.001** | **0.2 (0.1, 0.4)** | **0.002** |
| Gender |  |  |  |  |
| Female | Reference |  | Reference |  |
| Male | -4.4 (-14.7, 5.9) | 0.40 | 0.8 (-2.9, 4.5) | 0.66 |
| Education |  |  |  |  |
| Less than college | Reference |  | Reference |  |
| College | 5.6 (-10.3, 21.4) | 0.49 | -1.0 (-6.7, 4.7) | 0.72 |
| Greater than college | **16.3 (1.9, 30.8)** | **0.03** | 2.9 (-2.3, 8.1) | 0.28 |
| Race |  |  |  |  |
| White | Reference |  | Reference |  |
| Non-white | 1.1 (-9.8, 12.0) | 0.84 | 0.1 (-3.8, 4.0) | 0.95 |

| **Supplementary Table 4: Multiple Linear Regression of Deviation on the Triangle Completion Task and Card Rotations Test** | | | | |
| --- | --- | --- | --- | --- |
|  |  |  |  |  |
|  |  |  |  |  |
|  | **Distance of Deviation (cm)** | | **Angle of Deviation** | |
|  | **β (95% CI) ^a^** | **p-value** | **β (95% CI) ^a^** | **p-value** |
| Card Rotations Test | **-5.8 (-11.4, -0.2)** | **0.04** | -1.6 (-3.7, 0.5) | 0.12 |
| Age | **0.7 (0.3, 1.1)** | **0.001** | **0.2 (0.1, 0.4)** | **0.006** |
| Gender |  |  |  |  |
| Female | Reference |  | Reference |  |
| Male | -0.1 (-10.5, 10.2) | 0.98 | 1.5 (-2.3, 5.4) | 0.44 |
| Education |  |  |  |  |
| Less than college | Reference |  | Reference |  |
| College | 5.9 (-9.7, 21.5) | 0.46 | -0.6 (-6.4, 5.3) | 0.84 |
| Greater than college | **15.0 (0.8, 29.2)** | **0.04** | 3.3 (-2.0, 8.6) | 0.22 |
| Race |  |  |  |  |
| White | Reference |  | Reference |  |
| Non-white | -0.7 (-11.3, 10.0) | 0.90 | 0.6 (-3.4, 4.5) | 0.78 |

| **Supplementary Table 5: Multiple Linear Regression of Deviation on the Triangle Completion Task and Trail Making Test Part B (TMT-B)** | | | | |
| --- | --- | --- | --- | --- |
|  |  |  |  |  |
|  |  |  |  |  |
|  | **Distance of Deviation (cm)** | | **Angle of Deviation** | |
|  | **β (95% CI) ^a^** | **p-value** | **β (95% CI) ^a^** | **p-value** |
| TMT-B, seconds | **-5.9 (-11.3, -0.4)** | **0.03** | -1.6 (-3.5, 0.4) | 0.12 |
| Age | **0.7 (0.3, 1.1)** | **<0.001** | **0.2 (0.1, 0.4)** | **0.001** |
| Gender |  |  |  |  |
| Female | Reference |  | Reference |  |
| Male | -5.2 (-15.4, 5.0) | 0.31 | 0.6 (-3.1, 4.3) | 0.75 |
| Education |  |  |  |  |
| Less than college | Reference |  | Reference |  |
| College | 10.7 (-5.6, 26.9) | 0.20 | 0.4 (-5.5, 6.3) | 0.90 |
| Greater than college | **21.3 (6.6, 36.0)** | **0.005** | 4.3 (-1.1, 9.6) | 0.12 |
| Race |  |  |  |  |
| White | Reference |  | Reference |  |
| Non-white | 1.9 (-8.8, 12.7) | 0.72 | 0.6 (-3.3, 4.5) | 0.77 |

| **Supplementary Table 6: Multiple Linear Regression of Deviation on the Triangle Completion Task and Backward Digit Span, Wechsler Adult Intelligence Scale-Revised** | | | | |
| --- | --- | --- | --- | --- |
|  |  |  |  |  |
|  |  |  |  |  |
|  | **Distance of Deviation (cm)** | | **Angle of Deviation** | |
|  | **β (95% CI) ^a^** | **p-value** | **β (95% CI) ^a^** | **p-value** |
| Backward digit span | **-2.0 (-7.3, 3.3)** | 0.46 | -0.6 (-2.5, 1.3) | 0.53 |
| Age | **0.8 (0.4, 1.2)** | **<0.001** | **0.3 (0.1, 0.4)** | **<0.001** |
| Gender |  |  |  |  |
| Female | Reference |  | Reference |  |
| Male | -3.9 (-14.2, 6.4) | 0.46 | 1.0 (-2.7, 4.7) | 0.60 |
| Education |  |  |  |  |
| Less than college | Reference |  | Reference |  |
| College | 5.8 (-10.2, 21.9) | 0.48 | -0.9 (-6.7, 4.8) | 0.75 |
| Greater than college | **15.4 (1.2, 29.7)** | **0.034** | 2.7 (-2.4, 7.8) | 0.30 |
| Race |  |  |  |  |
| White | Reference |  | Reference |  |
| Non-white | 1.0 (-10.2, 12.2) | 0.86 | 0.0 (-4.0, 4.0) | 1.0 |

| **Supplementary Table 7: Multiple Linear Regression of Deviation on the Triangle Completion Task and Digit symbol substitution test** | | | | |
| --- | --- | --- | --- | --- |
|  |  |  |  |  |
|  |  |  |  |  |
|  | **Distance of Deviation (cm)** | | **Angle of Deviation** | |
|  | **β (95% CI) ^a^** | **p-value** | **β (95% CI) ^a^** | **p-value** |
| Digit symbol substitution test | **-10.9 (-17.7, -4.0)** | **0.002** | **-3.4 (-5.9, -0.8)** | **0.01** |
| Age | 0.3 (-0.2, 0.8) | 0.21 | 0.1 (-0.1, 0.3) | 0.32 |
| Gender |  |  |  |  |
| Female | Reference |  | Reference |  |
| Male | -9.4 (-19.7, 1.0) | 0.08 | 0.9 (-4.8, 2.9) | 0.63 |
| Education |  |  |  |  |
| Less than college | Reference |  | Reference |  |
| College | 4.3 (-10.9, 19.5) | 0.58 | -1.5 (-7.2, 4.2) | 0.61 |
| Greater than college | **15.5 (1.8, 29.2)** | **0.03** | 2.8 (-2.4, 7.9) | 0.29 |
| Race |  |  |  |  |
| White | Reference |  | Reference |  |
| Non-white | -2.8 (-13.5, 7.9) | 0.61 | -0.4 (-4.4, 3.6) | 0.83 |

| **Supplementary Table 8: Multiple Linear Regression of Deviation on the Triangle Completion Task and Letter Fluency test** | | | | |
| --- | --- | --- | --- | --- |
|  |  |  |  |  |
|  |  |  |  |  |
|  | **Distance of Deviation (cm)** | | **Angle of Deviation** | |
|  | **β (95% CI) ^a^** | **p-value** | **β (95% CI) ^a^** | **p-value** |
| Letter Fluency, mean | -4.8 (-10.0, 0.4) | 0.07 | -1.7 (-3.6, 0.2) | 0.08 |
| Age | **0.8 (0.4, 1.2)** | **<0.001** | **0.2 (0.1, 0.4)** | **0.001** |
| Gender |  |  |  |  |
| Female | Reference |  | Reference |  |
| Male | -3.8 (-13.8, 6.2) | 0.45 | 0.6 (-3.1, 4.3) | 0.73 |
| Education |  |  |  |  |
| Less than college | Reference |  | Reference |  |
| College | 7.6 (-8.1, 23.3) | 0.34 | -0.1 (-5.9, 5.7) | 0.97 |
| Greater than college | **17.3 (3.0, 31.5)** | **0.02** | 3.8 (-1.4, 9.1) | 0.15 |
| Race |  |  |  |  |
| White | Reference |  | Reference |  |
| Non-white | -0.7 (-11.3, 9.8) | 0.89 | -0.1 (-4.0, 3.8) | 0.97 |

| **Supplementary Table 9: Multiple Linear Regression of Deviation on the Triangle Completion Task and Category Fluency test** | | | | |
| --- | --- | --- | --- | --- |
|  |  |  |  |  |
|  |  |  |  |  |
|  | **Distance of Deviation (cm)** | | **Angle of Deviation** | |
|  | **β (95% CI) ^a^** | **p-value** | **β (95% CI) ^a^** | **p-value** |
| Category Fluency, mean | -4.1 (-10.5, 2.3) | 0.21 | -1.7 (-4.1, 0.6) | 0.15 |
| Age | **0.7 (0.2, 1.1)** | **0.004** | **0.2 (0.0, 0.4)** | **0.03** |
| Gender |  |  |  |  |
| Female | Reference |  | Reference |  |
| Male | -4.6 (-14.9, 5.7) | 0.38 | 0.3 (-3.5, 4.1) | 0.89 |
| Education |  |  |  |  |
| Less than college | Reference |  | Reference |  |
| College | 5.9 (-9.7, 21.5) | 0.46 | -0.6 (-6.4, 5.1) | 0.83 |
| Greater than college | **14.9 (1.0, 28.8)** | **0.04** | 3.1 (-2.1, 8.2) | 0.24 |
| Race |  |  |  |  |
| White | Reference |  | Reference |  |
| Non-white | -1.3 (-12.3, 9.7) | 0.81 | -0.4 (-4.4, 3.6) | 0.84 |

| **Supplementary Table 10: Multiple Linear Regression of Deviation on the Triangle Completion Task and Trail Making Test Part A (TMT-A)** | | | | |
| --- | --- | --- | --- | --- |
|  |  |  |  |  |
|  |  |  |  |  |
|  | **Distance of Deviation (cm)** | | **Angle of Deviation** | |
|  | **β (95% CI) ^a^** | **p-value** | **β (95% CI) ^a^** | **p-value** |
| TMT-A, seconds | -2.5 (-7.9, 3.0) | 0.37 | -0.6 (-2.5, 1.4) | 0.57 |
| Age | **0.8 (0.4, 1.2)** | **<0.001** | **0.3 (0.1, 0.4)** | **0.001** |
| Gender |  |  |  |  |
| Female | Reference |  | Reference |  |
| Male | -4.2 (-14.5, 6.1) | 0.42 | 0.9 (-2.8, 4.6) | 0.63 |
| Education |  |  |  |  |
| Less than college | Reference |  | Reference |  |
| College | 6.0 (-10.0, 22.0) | 0.46 | -0.9 (-6.7, 4.8) | 0.75 |
| Greater than college | **15.3 (1.1, 29.5)** | **0.03** | 2.6 (-2.5, 7.7) | 0.32 |
| Race |  |  |  |  |
| White | Reference |  | Reference |  |
| Non-white | 1.1 (-9.9, 12.1) | 0.84 | 0.1 (-3.8, 4.1) | 0.95 |

| **Supplementary Table 11: Multiple Linear Regression of Deviation on the Triangle Completion Task and Forward Digit Span, Wechsler Adult Intelligence Scale-Revised** | | | | |
| --- | --- | --- | --- | --- |
|  |  |  |  |  |
|  |  |  |  |  |
|  | **Distance of Deviation (cm)** | | **Angle of Deviation** | |
|  | **β (95% CI) ^a^** | **p-value** | **β (95% CI) ^a^** | **p-value** |
| Forward digit span | -2.6 (-7.9, 2.7) | 0.34 | -1.0 (-2.9, 0.9) | 0.32 |
| Age | **0.8 (0.4, 1.2)** | **<0.001** | **0.3 (0.1, 0.4)** | **<0.001** |
| Gender |  |  |  |  |
| Female | Reference |  | Reference |  |
| Male | -3.2 (-13.6, 7.2) | 0.55 | 1.2 (-2.5, 5.0) | 0.51 |
| Education |  |  |  |  |
| Less than college | Reference |  | Reference |  |
| College | 5.1 (-10.7, 21.0) | 0.52 | -1.1 (-6.8, 4.6) | 0.70 |
| Greater than college | **15.3 (1.2, 29.5)** | **0.03** | 2.7 (-2.4, 7.8) | 0.30 |
| Race |  |  |  |  |
| White | Reference |  | Reference |  |
| Non-white | 1.0 (-10.0, 12.0) | 0.86 | 0.0 (-4.0, 3.9) | 0.98 |

| **Supplementary Table 12: Multiple Linear Regression of Deviation on the Triangle Completion Task and Purdue Pegboard, Dominant Hand** | | | | |
| --- | --- | --- | --- | --- |
|  |  |  |  |  |
|  |  |  |  |  |
|  | **Distance of Deviation (cm)** | | **Angle of Deviation** | |
|  | **β (95% CI) ^a^** | **p-value** | **β (95% CI) ^a^** | **p-value** |
| Purdue Pegboard, Dominant | **-9.2 (-16.1, -2.3)** | **0.01** | -1.6 (-4.1, 1.0) | 0.22 |
| Age | 0.4 (-0.1, 0.9) | 0.13 | **0.2 (0.0, 0.4)** | **0.04** |
| Gender |  |  |  |  |
| Female | Reference |  | Reference |  |
| Male | -7.7 (-18.1, 2.8) | 0.15 | 0.2 (-3.7, 4.0) | 0.93 |
| Education |  |  |  |  |
| Less than college | Reference |  | Reference |  |
| College | 3.0 (-12.7, 18.6) | 0.71 | -1.6 (-7.3, 4.1) | 0.58 |
| Greater than college | 13.5 (-0.4, 27.5) | 0.06 | 2.3 (-2.8, 7.5) | 0.37 |
| Race |  |  |  |  |
| White | Reference |  | Reference |  |
| Non-white | 3.9 (-6.7, 14.5) | 0.47 | 0.8 (-3.1, 4.7) | 0.69 |

| **Supplementary Table 13: Multiple Linear Regression of Deviation on the Triangle Completion Task and Purdue Pegboard, Nondominant Hand** | | | | |
| --- | --- | --- | --- | --- |
|  |  |  |  |  |
|  |  |  |  |  |
|  | **Distance of Deviation (cm)** | | **Angle of Deviation** | |
|  | **β (95% CI) ^a^** | **p-value** | **β (95% CI) ^a^** | **p-value** |
| Purdue Pegboard, Nondominant | -5.1 (-12.0, 1.8) | 0.14 | -1.1 (-3.6, 1.4) | 0.39 |
| Age | **0.6 (0.1,1.1)** | **0.02** | **0.2 (0.0, 0.4)** | **0.02** |
| Gender |  |  |  |  |
| Female | Reference |  | Reference |  |
| Male | -4.3 (-14.7, 6.0) | 0.41 | 0.8 (-2.9, 4.5) | 0.67 |
| Education |  |  |  |  |
| Less than college | Reference |  | Reference |  |
| College | 6.2 (-9.8, 22.3) | 0.44 | -1.0 (-6.8, 4.8) | 0.74 |
| Greater than college | **17.6 (3.2, 32.0)** | **0.02** | 3.1 (-2.0, 8.3) | 0.23 |
| Race |  |  |  |  |
| White | Reference |  | Reference |  |
| Non-white | 2.3 (-8.6, 13.1) | 0.68 | 0.5 (-3.4, 4.4) | 0.82 |

| **Supplementary Table 14: Multiple Linear Regression of Deviation on the Triangle Completion Task and Purdue Pegboard, Mean** | | | | |
| --- | --- | --- | --- | --- |
|  |  |  |  |  |
|  |  |  |  |  |
|  | **Distance of Deviation (cm)** | | **Angle of Deviation** | |
|  | **β (95% CI) ^a^** | **p-value** | **β (95% CI) ^a^** | **p-value** |
| Purdue Pegboard, Mean | **-8.3 (-15.5, -1.1)** | **0.02** | -1.5 (-4.2, 1.1) | 0.25 |
| Age | 0.4 (-0.1, 0.9) | 0.15 | 0.2 (0.0, 0.4) | 0.06 |
| Gender |  |  |  |  |
| Female | Reference |  | Reference |  |
| Male | -5.6 (-16.0, 4.8) | 0.29 | 0.6 (-3.2, 4.4) | 0.77 |
| Education |  |  |  |  |
| Less than college | Reference |  | Reference |  |
| College | 5.0 (-10.7, 20.8) | 0.53 | -1.2 (-7.0, 4.5) | 0.67 |
| Greater than college | **16.2 (2.1, 30.2)** | **0.02** | 2.8 (-2.3, 8.0) | 0.28 |
| Race |  |  |  |  |
| White | Reference |  | Reference |  |
| Non-white | 2.9 (-7.8, 13.7) | 0.59 | 0.6 (-3.3, 4.5) | 0.77 |

| **Supplementary Table 15: Multiple Linear Regression of Deviation on the Triangle Completion Task and Mini Mental Status Examination (MMSE)** | | | | |
| --- | --- | --- | --- | --- |
|  |  |  |  |  |
|  |  |  |  |  |
|  | **Distance of Deviation (cm)** | | **Angle of Deviation** | |
|  | **β (95% CI) ^a^** | **p-value** | **β (95% CI) ^a^** | **p-value** |
| MMSE | -2.2 (-7.5, 3.2) | 0.42 | -0.3 (-2.2, 1.6) | 0.76 |
| Age | **0.8 (0.4, 1.2)** | **<0.001** | **0.3 (0.1, 0.4)** | **<0.001** |
| Gender |  |  |  |  |
| Female | Reference |  | Reference |  |
| Male | -5.1 (-15.6, 5.4) | 0.33 | 0.8 (-3.0, 4.5) | 0.69 |
| Education |  |  |  |  |
| Less than college | Reference |  | Reference |  |
| College | 5.7 (-10.3, 21.7) | 0.48 | -1.1 (-6.8, 4.7) | 0.71 |
| Greater than college | **15.9 (1.6, 30.2)** | **0.03** | 2.6 (-2.5, 7.8) | 0.31 |
| Race |  |  |  |  |
| White | Reference |  | Reference |  |
| Non-white | 2.6 (-8.2, 13.5) | 0.63 | 0.5 (-3.4, 4.4) | 0.81 |

| **Supplementary Table 16: Rotated Factor Loadings of Cognitive Tests: Baltimore Longitudinal Study of Aging** | | | |
| --- | --- | --- | --- |
|  |  |  |  |
|  |  |  |  |
| **Cognitive Test** | **Factor 1: Visuospatial Ability** | **Factor 2: Verbal Memory** | **Factor 3: Working Memory and Attention** |
|  |  |  |  |
| Mini Mental Status Examination | 0.2780 | **0.4449** | 0.1794 |
| Card Rotations test | **0.4178** | 0.2741 | 0.2088 |
| Purdue Pegboard |  |  |  |
| Dominant | **0.8601** | 0.1160 | -0.0284 |
| Nondominant | **0.8097** | 0.1016 | 0.0627 |
| BVRT, errors | **0.4394** | **0.3727** | **0.3364** |
| TMT-A , seconds | **0.4945** | 0.1871 | 0.1346 |
| TMT-B, seconds | **0.3980** | 0.2224 | 0.2609 |
| California verbal learning test |  |  |  |
| Immediate recall total | 0.2051 | **0.8632** | 0.1151 |
| Delayed recall | 0.1062 | **0.8631** | 0.0905 |
| Backward digit span | -0.0012 | 0.2280 | **0.6672** |
| Category Fluency, mean | **0.5622** | **0.5090** | 0.1490 |
| Letter Fluency, mean | 0.2694 | **0.3491** | 0.2361 |
| Forward digit span | 0.0241 | 0.1075 | **0.6198** |
| Digit symbol substitution test | **0.7261** | **0.3138** | 0.0388 |
|  |  |  |  |
| Loading > 0.3 in bold | | | |
